# Modulation of Active Gut Microbiota by *Lactobacillus rhamnosus* GG in a Diet Induced Obesity Murine Model

**DOI:** 10.3389/fmicb.2018.00710

**Published:** 2018-04-10

**Authors:** Yosep Ji, Soyoung Park, Haryung Park, Eunchong Hwang, Hyeunkil Shin, Bruno Pot, Wilhelm H. Holzapfel

**Affiliations:** ^1^Graduate School of Advanced Green Energy and Environment, Handong Global University, Pohang, South Korea; ^2^School of Life Sciences, Handong Global University, Pohang, South Korea; ^3^Research Group of Industrial Microbiology and Food Biotechnology, Department of Bioengineering Sciences, Vrije Universiteit Brussel, Brussels, Belgium

**Keywords:** 16S rDNA analysis, 16S rRNA analysis, gut microbiota, obesity, LGG

## Abstract

Gut microbiota play a key role in the development of metabolic disorders. Defining and correlating structural shifts in gut microbial assemblages with conditions related to metabolic syndrome have, however, been proven difficult. Results from 16S genomic DNA and 16S ribosomal RNA analyses of fecal samples may differ widely, leading to controversial information on the whole microbial community and metabolically active microbiota. Using a C57BL/6J murine model, we compared data from 16S genomic DNA and ribosomal RNA of the fecal microbiota. The study included three groups of experimental animals comprising two groups with high fat diet induced obesity (DIO) while a third group (control) received a low fat diet. One of the DIO groups was treated with the probiotic *Lactobacillus rhamnosus* GG (LGG). Compared to the data obtained by DNA analysis, a significantly higher abundance of OTUs was accounted for by RNA analysis. Moreover, rRNA based analysis showed a modulation of the active gut microbial population in the DIO group receiving LGG, thus reflecting a change in the induced obesity status of the host. As one of the most widely studied probiotics the functionality of LGG has been linked to the alleviation of metabolic syndrome, and, in some cases, to an impact on the microbiome. Yet, it appears that no study has reported thus far on modulation of the active microbiota by LGG treatment. It is postulated that the resulting impact on calorie consumption affects weight gain concomitantly with modulation of the functional structure of the gut microbial population. Using the 16S rRNA based approach therefore decisively increased the precision of gut microbiota metagenome analysis.

## Introduction

Application of novel techniques such as those based on culturomics has revealed a hitherto unexpected complexity of the human gut microbiome; numerous microbial taxonomic units have only recently been detected, bringing the estimated number of microbial species to more than 1500 at present (Lagier et al., 2016; Utzschneider et al., 2016). The microbiome of the human gastrointestinal tract (GIT) varies among healthy individuals (Human Microbiome Project Consortium, 2012; Yatsunenko et al., 2012), but its overall balance will decisively influence the functioning of the metabolic, mental and immune systems of the host (Nicholson et al., 2012; Sanz et al., 2015; Ussar et al., 2015; Liu, 2017). Bäckhed et al. (2004) have shown earlier that gut microbiota serve as an environmental factor to regulate fat storage. While shifts or alterations in the autochthonous microbial population of people suffering from metabolic diseases have been reported frequently (Turnbaugh et al., 2006; Cani et al., 2008; Raoult, 2008; Armougom et al., 2009; Cani and Delzenne, 2009; Larsen et al., 2010; Zhang et al., 2013; Kasai et al., 2015; Ussar et al., 2015), this issue has, however, remained controversial, since an overwhelming part of the data has been acquired by microbial genomic DNA- and not ribosomal RNA-based gut microbiota analysis. The latter technology may reflect more reliably the metabolically active microbial communities more precisely (Baldrian et al., 2012; Pérez-Cobas et al., 2013).

Elucidating metabolically active microbial communities within the complexity of the total microbiota constitutes an essential yet intriguing challenge toward comprehending the role of commensal microbiota in health and disease. Peris-Bondia et al. (2011) applied flow cytometry to isolate the active bacterial fraction from human feces and found a huge part of the active microbiota to be statistically neglected or “overshadowed” by the enormous diversity of the total microbiota. Using both 16S rDNA and 16S rRNA analyses of fecal samples, Pérez-Cobas et al. (2013) found meaningful differences between the whole microbiota composition and the active fraction of the microbiota in an antibiotic treated patient. The paper suggested only a minor part of the microbial community plays a significant role in terms of their metabolic activity, independent of the complexity of the whole community. Moreover, the impact of sample processing procedures on the results has been confirmed by Walker et al. (2015) when using cell disruption methods for DNA extraction in 16S rRNA gene profiling of infant gut microbiota in conjunction with optimized ‘universal’ PCR primers. Using mock communities and mock community DNA, Fouhy et al. (2016) showed the importance on 16S rRNA sequencing results of the sequencing platform besides the DNA extraction method and the primer sequences used. Contradictory results of rRNA amplicon-based metagenome analysis have been reported by Blazewicz et al. (2013), showing no clear correlation of the rRNA of different groups of microorganisms with their growth and activity, potentially leading to exaggerated and/or underestimated expression of metabolic activeness of microbiota. Relative to the functional potential of an ecosystem, a dynamic interplay continues between growing, metabolically active and dormant cells. Studying the same rumen microbiota using RNA-seq and RNA/DNA amplicon-seq methods revealed differences in taxonomic profiles (Li et al., 2016), but suggested a higher robustness of RNA-based methods for detecting microbial phylotypes with potential metabolic activities. Similarly, compared to DNA amplicon-seq, RNA-based phylotypes revealed more interactions and a higher diversity (Li et al., 2016). Without any doubt, more reliable information may be obtained by duplicate parallel studies comparing control and test groups concomitantly with gDNA and rRNA information.

This study was conducted to compare DNA vs. RNA approaches for gut microbiota analysis in a C57BL/6J mouse model by modifying the gut ecosystem system with a specific high fat diet (control: low fat diet) and by the administration of the probiotic strain *Lactobacillus rhamnosus* GG (LGG).

## Materials and Methods

### Bacterial Strains and Culture Conditions

*Lactobacillus rhamnosus* GG (LGG) was grown in MRS broth (Difco Laboratories Inc., Franklin Lakes, NJ, United States) and prepared daily for feeding during the intervention period. LGG was grown for 8 h to reach the late log phase, collected (16,000 ×*g*, 5 min, 4°C) and washed two times with PBS. The approximate viable numbers were adjusted with PBS to 1 × 10^7^ CFU/ml using a standard curve (data not shown). Optical density was determined using a SPECTROstar Nano (BMG Labtech, Durham, NC, United States) spectrophotometer. The prepared sample was suspended in 200 μl of PBS for daily oral administration by gavage.

### Study Design and Animals

The animal study was approved by the Handong Global University ethical committee in South Korea (Ethical Committee No. 20151022-009). Seven week old, specific pathogen free (SPF) male C57BL/6J mice were supplied by Hyochang Science, Daegu, South Korea. The animals were housed at 23°C and 55 ± 10% humidity, in a 12 h light/dark cycle. After 1 week of adaptation, 20 mice were separated into three different groups (6 for the low fat diet control and 7 for the other groups), receiving different treatments (Supplementary Figure S1 and **Table 1**). The high fat diet (Research Diets D12492, New Brunswick, NJ, United States) (HFD) and autoclaved tap water were provided *ad libitum* to induce obesity, while the low fat diet (Research Diets D12450, New Brunswick, NJ, United States) (LFD) was provided to a control group. For a period of 10 weeks the HFD mice were orally administered daily either phosphate buffered saline (PBS) or LGG suspended in PBS (1 × 10^7^ CFU/day). The weight of each animal and its feed consumption were measured once a week. Three representative mice from each group were further analyzed for their fat mass using Micro-PET/CT (Siemens Preclinical Solution, Knoxville, TN, United States), using Inveon software. Body fat content was measured using the micro-CT method 1 week prior to sacrifice. On the last day of the experiment, mice were sacrificed by cervical dislocation. After the study, the serum, small intestine, colon, liver, epididymal adipose tissue, and spleen of each experimental animal were collected, weighed and kept at -80°C until analysis. Fecal samples were collected within 6 h after changing the bedding of the cages and half of the fecal samples were immediately immersed into RNAlater (Thermo Scientific, Waltham, MA, United States) to avoid deterioration of microbial RNA. In a metagenomic approach, genomic DNA and ribosomal RNA, were extracted from faecal samples of each group, and used to compare the whole community (DNA) and the metabolically active community structure (RNA) of the fecal microbiota.

**Table 1:** Composition of experimental groups receiving high-fat (HFD) and low-fat diet (LFD), and high-fat diet with the probiotic strain *L. rhamnosus* GG.

Group	Feed	Treatment
LFD (*n* = 6)	LFD	200 μL PBS only (non-obese control)
HFD (*n* = 7)	HFD	200 μL PBS only (obese control)
HFD+LGG (*n* = 7)	HFD	1 × 10^7^ CFU/day of LGG suspended in 200 μL of PBS

### Serum Analysis

Multiplex sandwich ELISAs were performed using the Magnetic Luminex Screening Assay (Koma Biotechnology, Seoul, South Korea) to monitor adiponectin, leptin, and interleukin 12 p70 (IL-12p70) from a single serum sample of each individual mouse.

### RNA Extraction and Reverse Transcription of Organs

Extraction of mRNA from epididymal adipose tissue followed the RNeasy protocol of the RNA tissue miniprep system (Promega, United States). Briefly, each organ sample was homogenized by a hand-held homogeniser (IKA, Germany) in a lysis buffer and centrifuged. Supernatant was mixed with isopropanol and passed through a column provided with the kit. After several washing and DNase treatments, the purity and concentration of the eluted RNA was measured by a SPECTROstar (BMG LABTECH, Germany) spectrophotometer. 2–3 μg of complement DNA (cDNA) was prepared using the GoScript^TM^ Reverse Transcription System (Promega, United States) using a Verity 96-well thermal cycler (ABI research, United States) after 10 min of incubation with oligodT primer at 70°C.

### Quantitative Real-Time PCR (qRT-PCR) Analysis

The ABI 7500 Real-Time PCR System (Applied Biosystems, Carlsbad, CA, United States) and SYBR Premix Ex Taq RR420 (TaKaRa, Japan) was used for qRT-PCR, following the protocols previously described (Ji et al., 2012). The Delta Delta *C*(t) method (Livak and Schmittgen, 2001) was used to calculate gene expression levels of different biomarkers; the primers are listed in Supplementary Table S1.

### Extraction of Microbial Genomic DNA and Ribosomal RNA for Metagenomic Analysis

Genomic DNA (DNA) and ribosomal RNA (RNA) were extracted from individual faecal microbiota samples using the ReliPrep gDNA Tissue Miniprep System as well as the RNA Tissue Miniprep System (Promega, United States), after mechanical disruption of microbial cell walls. Briefly, for fecal rRNA extraction and preparation of cDNA, 50 mg of fecal sample were suspended in 500 μL of lysis buffer solution (490 μL of LBA buffer + 10 μL of 1-thioglycerol) in a screw cap micro-tube (Sarstedt, Germany) together with 0.3 g of 0.1 mm zirconium/silica beads (Biospec, United States), followed by disruption in a mini-beadbeater-16 (Biospec, United States) for 2 min and centrifuged at 14,000 ×*g* for 3 min at room temperature. The supernatant was mixed with 150 μL of isopropanol and transferred to ReliaPrep Minicolumn after vigorously mixing for 10 s. Subsequent clean up and DNase treatment procedures precisely followed the manufacturer’s instructions. RNA was finally eluted in a column using 30 μL of elution buffer, and further reverse transcribed to cDNA after measuring purity and concentration of the eluted RNA by SPECTROstar (BMG LABTECH, Germany); 1 μg of RNA was mixed with 1 μL of Random Primers (Promega, United States) and incubated at 70°C for 10 min and immediately cooled on ice for 10 min. Random Primer attached RNA was mixed with GoScript Reverse Transcription System master mix (Promega, United States) comprising 4 μL of GoScript 5X Reaction Buffer, 4 μL of MgCl_2_ (25 mM), 1 μL of PCR Nucleotide Mix, 1 μL of GoScript Reverse Transcriptase and 4 μL of Nuclease-Free Water. The PCR procedure was continued following the manufacturer’s instructions. For fecal bacterial gDNA extraction 50 mg of fecal sample were suspended in 720 μL of lysis buffer solution (320 μL of PBS + 400 μL of CLD) in a screw cap micro-tube (Sarstedt, Germany) together with 0.3 g of 0.1 mm zirconium/silica beads (Biospec, United States), followed by disruption in a mini-beadbeater-16 (Biospec, United States) for 2 min and centrifuged at 14,000 ×*g* for 3 min at room temperature. The supernatant was thoroughly mixed with 250 μL of Binding Buffer (BBA) and placed on a binding column. For the further cleaning and eluting process the manufacturer’s instructions were followed.

### Gut Microbiota Analysis Using 454 GS FLX and Bioinformatics Analysis

Gut microbial metagenome analysis was performed with the Roche 454 GS FLX plus system (AtoGen, Daejeon, South Korea) using genomic DNA (gDNA) and cDNA templates produced from reverse transcription of ribosomal RNA of the microbiota. Briefly, forward and reverse primers were designed based on V1–V3 variable region of the 16S rDNA sequence (forward, 8f: 5′-AGAGTTTGATCMTGGCTCAG-3′; reverse 518r: 5′-ATTACCGCGGCTGCTGG-3′) and tagged with10 bp unique barcode labels (Supplementary Table S2).

Flow pattern B raw data from the 454 GS FLX plus system (Roche, Switzerland) was denoised and filtered using FlowClus (Gaspar and Thomas, 2015) and further analyzed using the MacQIIME (Caporaso et al., 2010) 1.8.0 pipeline. Briefly, chimeras were eliminated using Usearch 6.1 and sequences were clustered into operational taxonomic units (OTU) at 99% sequence similarity, and taxonomically assigned using the RDP database. A total of 276 199 raw reads were produced comprising an average of 15 344 reads per sample and an average read length of 456 nucleotides per sample. 209 859 of the reads were selected after excluding sequencing noise and possible chimeras, yielding 10 878 useable reads per sample on average (Supplementary Figure S2).

Composition and intra-/inter- community diversity (alpha and beta diversity) of taxonomically assigned microbiota were interpreted using two-dimensional (2D) and three-dimensional (3D) weighted principal coordinates analysis (PCoA) and 2D weighted pair group method averaging (WPGMA; the WPGMA dendrogram is presented in **Figure 4C**). Distance matrix was also performed to understand the degree of gut microbiota differences. Post analysis including alpha, beta diversity and taxonomic assignment of microbiota was conducted using a Qiime pipeline (Caporaso et al., 2010). Metagenome information is deposited in the Sequence Read Archive of the NCBI under accession number of PRJNA342544.

### Short Chain Fatty Acid Analysis

Short chain fatty acids (SCFA) analysis was performed according to the method of Schwiertz et al. (2010). Briefly, 50 mg of deep-frozen caecum was mixed with 500 μL of extraction solution (comprising 100 mmol oxalic acid /l and 40 mmol sodium azide /l), incubated on a horizontal shaker for an hour at room temperature, and centrifuged at 16 000 ×*g* for 10 min. The supernatant was filtered through a 0.45 μm Minisart RC 4 syringe filter (Sartorius Stedim Biotech, Germany), transferred to a Clear gas chromatography vial (Shimadzu, United States) and tightly sealed using a Ribbed blue screw vial cap with bonded silicone (Shimadzu, United States) until analysis. A GC-2010 (Shimadzu, Japan) and HP-Innowax 30 m × 0.32 mm × 0.25 μm column (Agilent, United States) were used for detection; N_2_ gas served as carrier gas. 1 μL of each sample was injected by Shimadzu Auto-sampler AOC-20is (Shimadzu, Japan) at 260°C and detected by a flame ionized detector (FID). The column temperature was increased from 100°C up to 180°C at a rate of 25°C/min. A volatile free acid standard mix (Supelco, United States) was used as analytical standard of C2 through C5.

### Statistical Analysis

All the graphs are presented as mean value and standard error (SD). GraphPad Prism 6.0 (GraphPad Software, United States) was used for non-parametric one-way and two-way analysis of variance (ANOVA) and *post hoc* multiple comparisons were followed using Bonferroni’s multiple comparison test. IBM SPSS statistics version 20 (IBM, United States) was used to calculate the Pearson correlation coefficient and the accepted significance level of correlation curves. All the significance was accepted at *P* < 0.05 and indicated as various symbols as ^∗^, $, and # (<0.05 = one symbol; <0.01 = two symbols and <0.001 = three symbols).

## Results

### Physiological Impact

**FIGURE 1:**
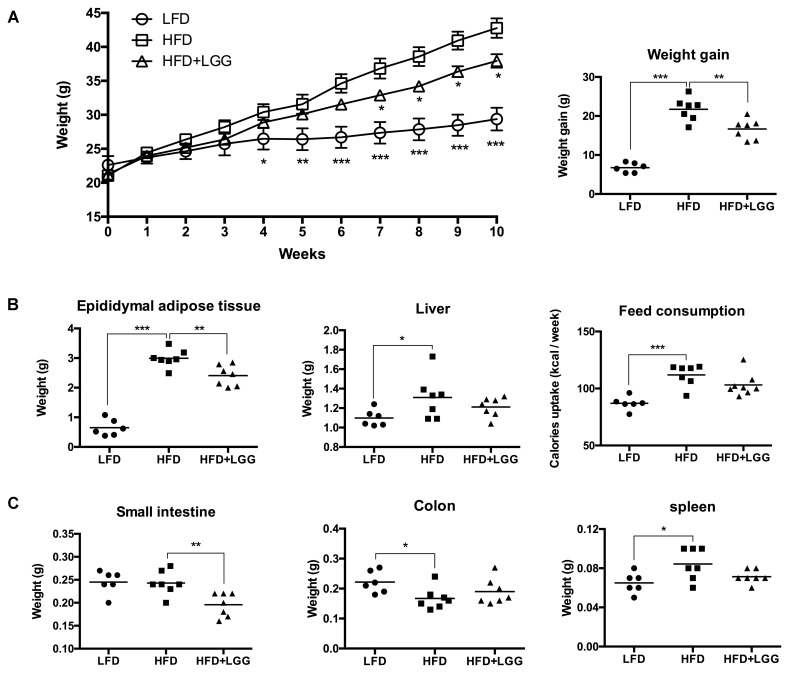
Impact on body and organ weight by HFD and LGG in a DIO murine model, showing **(A)** weekly weight changes and total weight gain. **(B)** EAT, liver and feed consumption. **(C)** Small intestine, colon and spleen weight. Significance level was calculated using ANOVA and Bonferroni’s multiple comparisons test. Results are illustrated as ^∗^*p* < 0.05, ^∗∗^*p* < 0.01, ^∗∗∗^*p* < 0.001.

**FIGURE 2:**
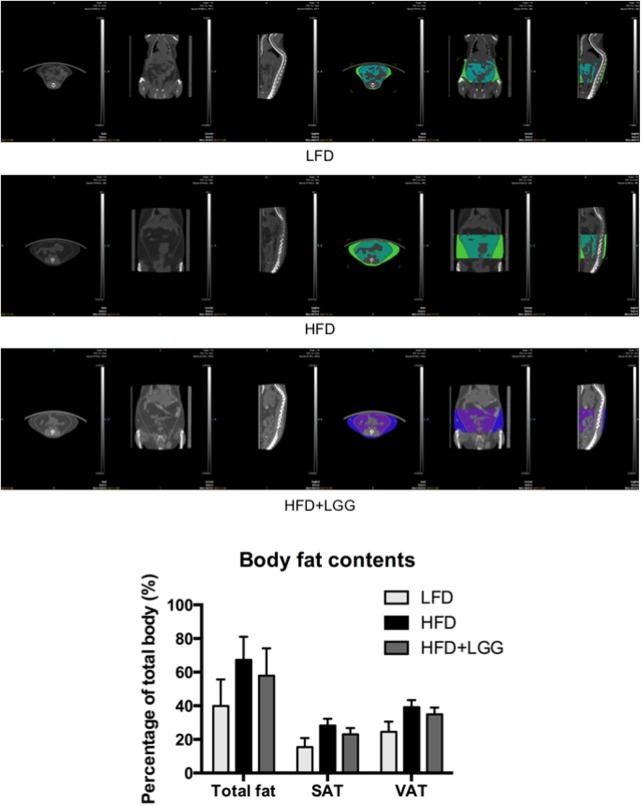
Pictures of micro-CT of each group. Fat mass was measured between lumbar numbers 1–6; visceral adipose tissue (VAT) and subcutaneous adipose tissue (SAT) were visualized and measured in different colors. Fat mass was first calculated in volume (mm^3^) to indicate its percentage out of total body volume using Inveon software.

**FIGURE 3:**
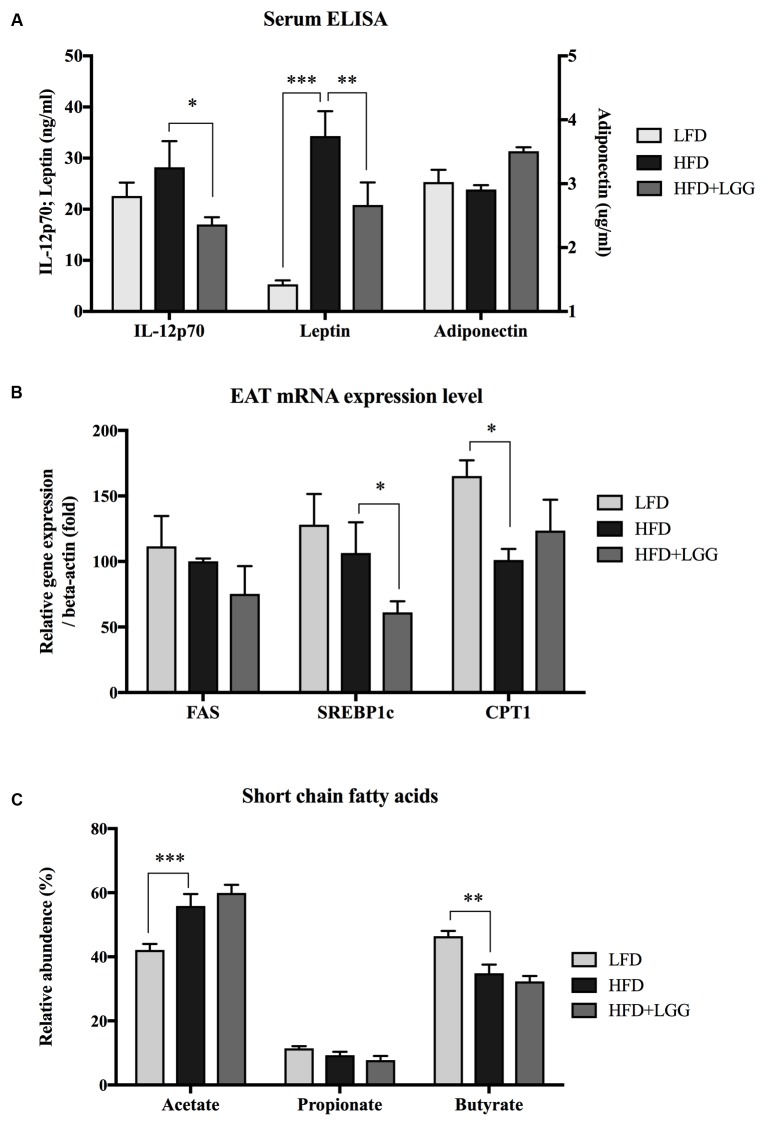
**(A)** Serum IL-12p70, leptin and adiponectin were measured using the ELISA method. **(B)** mRNA expression level of fatty acid synthase (FAS), sterol regulatory element-binding protein 1c (SREBP1c) and carnitine palmitoyltransferase 1 (CPT1) were measured from epididymal adipose tissue (EAT). **(C)** Short chain fatty acids from fecal samples of each group were measured by gas chromatography. Significance level was calculated by using ANOVA and Bonferroni’s multiple comparisons test; results are illustrated as ^∗^*p* < 0.05, ^∗∗^*P* < 0.01, ^∗∗∗^*P* < 0.001.

Administration of a HFD for 10 weeks induced a significantly higher weight gain compared to the LFD control group (**Figure 1A**), and was associated with a significantly lower calorie uptake in this group (**Figure 1B**). Compared to the HFD PBS-treated group, weight gain was significantly lower in the probiotic LGG treatment group (HFD+LGG) (**Figure 1A**) while a minor reduction in calorie uptake was detected in the HFD+LGG group. Both the liver and epididymal adipose tissue (EAT) weight was significantly lower in the LFD group, while only the EAT weight was significantly reduced in the HFD+LGG group (**Figure 1B**). Both total body fat as well as subcutaneous adipose tissue (SAT) and visceral adipose tissue (VAT) were non-significantly reduced between lumbar one and six of three representative mice from each group (**Figure 2**). A significant decrease in weight of the small intestine was measured in the HFD+LGG group while the weight of the colon was significantly increased in the LFD group. The spleen weight of LFD animals was slightly lower than that of the HFD+LGG group but significantly lower than that of the HFD group (**Figure 1C**). Serum immune-metabolic biomarkers such as IL-12p70 and leptin were significantly reduced in the HFD+LGG group compared to the HFD group, while adiponectin was noticeably higher in the HFD+LGG group compared to the HFD group (**Figure 3A**). Sterol regulatory element-binding protein 1c (SREBP1c) was significantly down-regulated in the HFD+LGG group relative to the HFD group while lipid oxidation associated carnityl palmitoyltransferase 1 (CPT1) gene expression was only significantly up-regulated in the LFD group compared to the HFD group (**Figure 3B**). Among the SCFAs, significantly lower acetate and higher butyrate values were detected in fecal samples of the LFD group relative to the HFD and HFD+LGG groups (**Figure 3C**).

### Alpha and Beta Diversity of the Murine Microbiota

**FIGURE 4:**
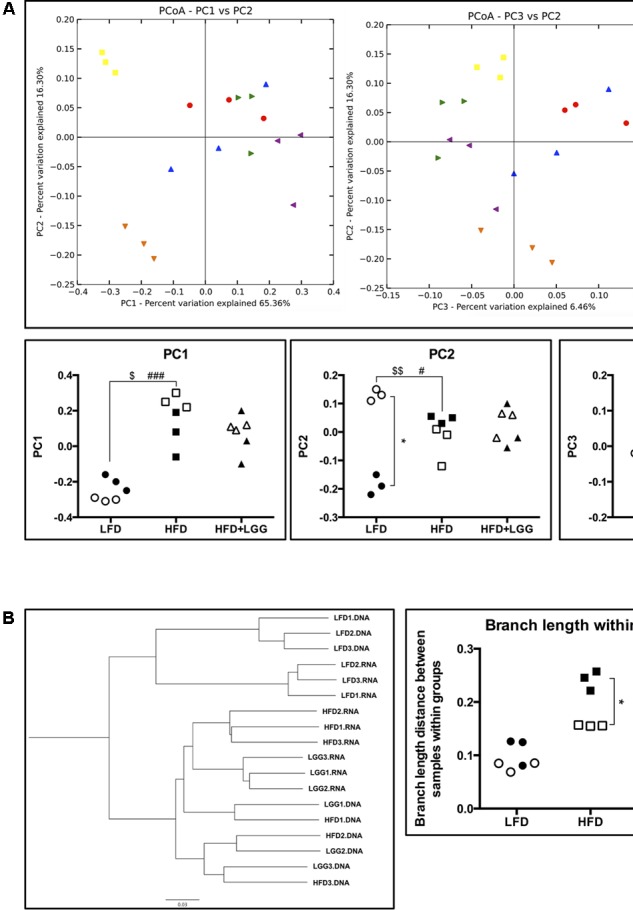
Alpha- and beta-diversity of fecal microbiota in a murine DIO model. **(A)** Weighted principal coordinates analysis and value of each principal component dimensions; **(B)** weighted pair group method with averaging and calculated branch length of samples within each group. Significance level was calculated using ANOVA and Bonferroni’s multiple comparisons test; ^∗^ shows significant difference between DNA and RNA analysis, $ shows significant difference of LFD and HFD+LGG compared to HFD according to DNA analysis, and # shows significant difference of LFD and HFD+LGG compared to HFD according to RNA analysis. The level of significance is expressed by the number of symbols (one = <0.05, two = <0.01, three = <0.001).

PD whole-tree rarefaction analysis showed lower alpha diversity in both gDNA and rRNA analyses when LFD was applied as compared to the HFD group (Supplementary Figure S3). Beta diversity of weighted principal coordinates analysis (PCoA) indicated a major impact of the different diets (LFD and HFD) on the associated microbiota (**Figure 4A**). PC1 of PCoA significantly reflected microbiota differences under different diets regardless of whole community (DNA) or active community (RNA) analysis. On the other hand, PC2 showed a strong difference between DNA and RNA analysis in the LFD group, implying that the active microbial community under LFD feeding can be interpreted differently from a whole community structure of the microbiome. With regard to PC3 of PCoA, significantly different DNA and RNA results were obtained in the HFD and HFD+LGG groups, suggesting differences in the impact of feeding regimes on microbial groups (PC2 vs. PC3). Significantly shorter branch lengths were measured in the RNA analysis of the HFD and HFD+LGG groups (**Figure 4B**).

### Taxonomic Summary of Microbiota

**FIGURE 5:**
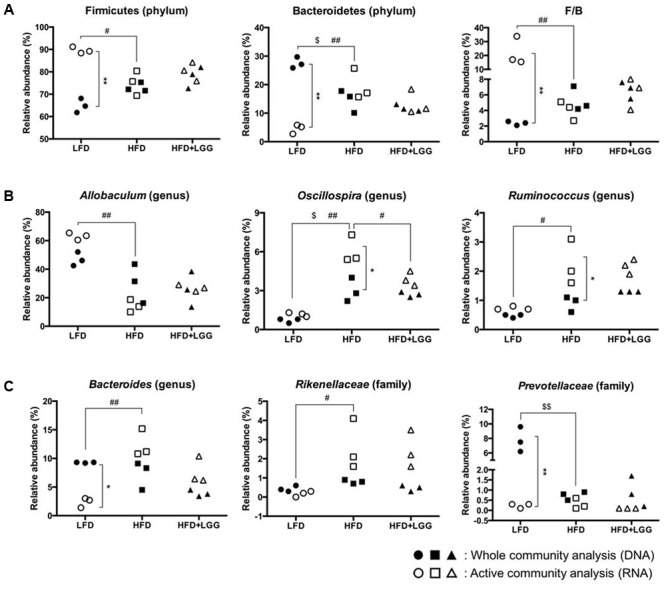
Fecal microbiota of LFD, HFD, and HFD+LGG groups. Black colored circle, rectangle, and triangle illustrate results of DNA analysis while empty symbols illustrate results of RNA analysis. **(A)** *Firmicutes, Bacteroidetes*, and *Firmicutes* over *Bacteroidetes* ratio (F/B); **(B)** major sub-groups of *Firmicutes*; **(C)** major sub-groups of *Bacteroidetes*. Significance level was calculated using ANOVA and Bonferroni’s multiple comparisons test and ^∗^ shows significant difference between DNA and RNA analysis, $ shows significant difference of LFD and HFD+LGG compared to HFD according to DNA analysis and # shows significant difference of LFD and HFD+LGG compared to HFD according to RNA analysis. The level of significance is expressed by the number of symbols (one = <0.05, two = <0.01, three = <0.001).

Fourteen major groups were identified at genus level, excluding minor groups representing <1% of the total microbiota. The Firmicutes and Bacteroidetes and their sub-groups comprised the major components of the microbiota. Bar graph based taxonomic summaries at phylum, class, family, and genus levels (Supplementary Figure S4) show major differences between LFD and HFD for Firmicutes and Bacteroidetes, depending on whether DNA or RNA analysis of microbiota has been applied. DNA analysis revealed a lower Firmicutes level in the LFD group compared to the HFD group while, by contrast, RNA analysis resulted in significantly higher Firmicutes levels mostly associated with OTU numbers of Clostridiales, *Lachnospiraceae*, and *Allobaculum* (Supplementary Figure S4). Based on DNA analysis *Bacteroidetes* levels were higher in the LFD group compared to the HFD group but significantly lower according to RNA analysis, resulting in a contradictory outcome for the Firmicutes: Bacteroidetes ratio (F/B) (**Figure 5A**). *Allobaculum, Oscillospira*, and *Ruminococcus* were found to be the major genera in the *Firmicutes*, with RNA analysis showing significantly higher *Allobaculum* and significantly lower *Ruminococcus* levels in the LFD group. Both DNA and RNA analyses showed significantly lower levels for *Oscillospira* in the LFD group while only RNA analysis could reveal a significant reduction of *Oscillospira* in the HFD+LGG group; this may be attributed to the significantly higher abundance of OTUs accounted for by RNA analysis (**Figure 5B**). *Bacteroides*, *Rikenellaceae*, and *Prevotellaceae* represented the major groups under the phylum *Bacteroidetes*. Both *Bacteroides* and *Prevotellaceae* showed significantly lower abundance of OTUs by RNA analysis as compared to DNA analysis in the LFD group. Significantly higher levels of *Bacteroides* and *Rikenellaceae* were detected in the HFD group compared to the LFD group, but only DNA analysis showed significantly higher abundance of *Prevotellaceae* in the LFD group (**Figure 5C**). The level of Lactobacillales was noticeably higher in the HFD+LGG group as compared to the HFD group, while the *Lachnospiraceae* and *Desulfovibrionaceae* were not significantly modulated (Supplementary Figure S3).

### Active and Whole Community of Microbiota

**FIGURE 6:**
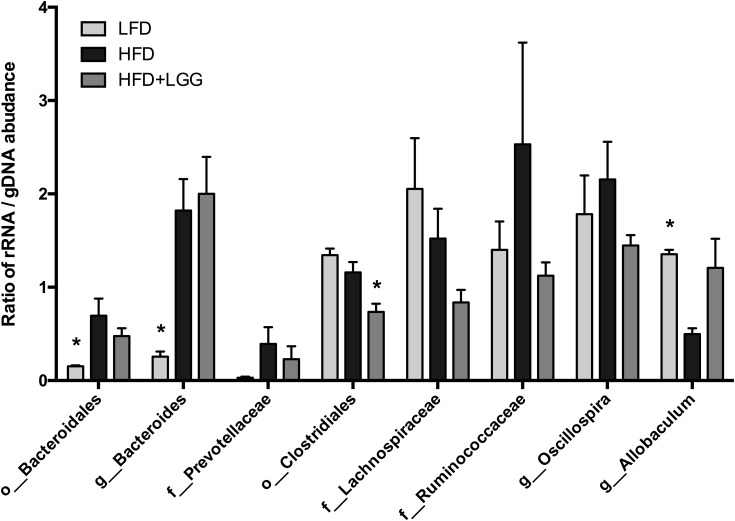
Ratio of rRNA/gDNA abundance in the microbiome of LFD, HFD, and HFD+LGG groups. Significance level was calculated by using ANOVA and Bonferroni’s multiple comparisons test; results are illustrated as ^∗^*p* < 0.05, ^∗∗^*p* < 0.01, ^∗∗∗^*p* < 0.001.

**FIGURE 7:**
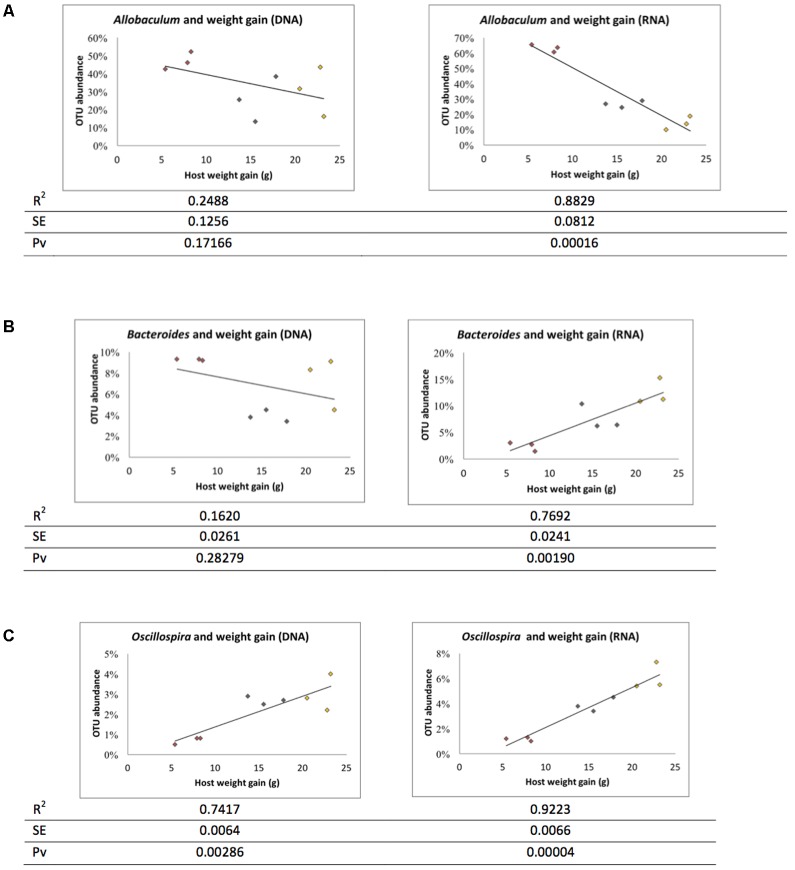
Correlation between microbiota and weight gain. Correlation graph and R square (*R*^2^), standard error (SE) and Pearson correlation coefficient *p*-value (Pv) were derived based on weight gain. **(A)** *Allobaculum*, **(B)** *Bacteroidetes*, and **(C)** *Oscillospira*. Red: LFD; orange: HFD; black: HFD+LGG.

The ratio of active and whole community microbiota was calculated among major groups and the LFD group was significantly correlated with a reduced ratio of *Bacteroides* species and an increase in *Allobaculum* species compared to the HFD group (**Figure 6**). The HFD+LGG group was associated with a reduced Clostridiales population compared to the HFD group. Correlation curves in **Figures 7A–C** show a significant relationship between weight gain (*X*-axis) and the genera *Allobaculum*, *Bacteroides*, and *Oscillospira* (*Y*-axis). *R*^2^ results and *p*-values obtained with the Pearson correlation coefficient indicate *Allobaculum* to be negatively and *Oscillospira* positively correlated with weight gain, implying a differential influence of diet on sub-groups within the Firmicutes and/or respective differences in the influence of such groups on the host physiological status. However, while DNA based data for Bacteroides suggest a non-significant correlation with weight gain, the RNA based data show a significantly positive correlation with weight gain (**Figure 7B**).

## Discussion

Microbiota plays an essential role in host energy metabolism and it constitutes a complex ecosystem averaging 70 different bacterial divisions that colonize the gut of an adult (Gérard, 2016). However, the specific number and activities of these microbes vary with regard to environmental factors; in fact, the metabolic status of these microbial groups may range from active to dormant, this being crucial in understanding and explaining the role of gut microbiota (Blazewicz et al., 2013). Our study compared the microbiome of high fat diet induced obese mice with or without probiotics using microbial rRNA and gDNA in order to gain a deeper understanding of those microbiota that are actively associated with a high fat diet and modulated by probiotic treatment. Ribosomal RNA comprises more than 80% of the total RNA with the overall amount of RNA correlating with growth and activity of microbes (Neidhardt and Magasanik, 1960; Kramer and Singleton, 1992; Tolker-Nielsen et al., 1997; Ramos et al., 2000). This basic principle was widely applied in various studies to define the existence of active microbiota in an environment (Wüst et al., 2011; Hunt et al., 2013; Männistö et al., 2013). Based on these considerations, we have designed our experiments to reveal specific information on metabolically active groups of the gut microbiome in a diet induced obesity (DIO) murine model and under application of the widely studied probiotic LGG.

The impact of GIT microbiota on host energy metabolism has been widely studied in recent years. However, a key, albeit a controversial issue, is to define core microbial assemblages associated with host weight gain and adipose tissue accumulation. This is underpinned by the rapid increase in body fat mass of germ-free mice colonized with gut microbiota from conventionally housed mice, in spite of decreased food consumption (Bäckhed et al., 2004). Even more interestingly, a greater weight increase in germ-free mice after colonization with microbiota from obese mice as compared to experimental animals colonized with microbiota from lean mice has also been reported (Turnbaugh et al., 2006). It appears indisputable that host gut microbiota exercise leverage over energy efficiency and adipose tissue accumulation, as has also been supported by gnotobiotic mice studies (Cani et al., 2008; Delzenne et al., 2011; Greiner and Bäckhed, 2011).

Recently, specific bacterial genera such as *Akkermansia* (Shin et al., 2014) and *Faecalibacterium* (Miquel et al., 2015) were reported as microbial groups important to host health, with e.g., *Akkermansia muciniphila* numbers found to be inversely correlated with inflammatory conditions and metabolic disorders in mice (Schneeberger et al., 2015). Based on findings showing *Bacteroidetes* not to be correlated with host weight gain, Ley et al. (2005) first proposed a possible correlation of the Firmicutes: Bacteroidetes ratio with propensity of host obesity. Yet, conflicting reports are increasingly pointing toward the role of the *Bacteroidetes* phylum, or its sub-groups, in influencing host energy efficiency (Larsen et al., 2010; Semova et al., 2012). DNA analysis in our study showed that the *Bacteroidetes* (in particular *Bacteroides* spp.) in the GIT was not correlated with host weight gain under a high fat diet (HFD) while, in contrast, a significantly positive correlation was found based on metabolic activity (RNA based data) of this phylum when compared with a low fat diet (LFD). The Firmicutes represents a vastly diverse phylum, comprising the three different classes Bacilli, Clostridia, and Erysipelotrichia (apart from the cell-wall-less Mollicutes). Our data show opposite roles for *Clostridia* (*Oscillospira* spp.) and *Erysipelotrichia* (*Allobaculum* spp.) associated with host weight. *Erysipelotrichaceae* and *Allobaculum* appear to represent the key family and genus, respectively, of the class *Erysipelotrichia* of relevance to host metabolic disorders. This issue is extensively discussed in various articles including a review article by Kaakoush (2015), who also refers to conflicting reports resulting in contradictions in the definition of the role of the *Erysipelotrichia* in host metabolic disorders. Briefly, Turnbaugh et al. (2008) and Zhang et al. (2009) reported increasing numbers of *Erysipelotrichaceae* in diet induced obese mice and in obese individuals, while Ravussin et al. (2012) and Everard et al. (2014) found *Allobaculum* to be negatively correlated with DIO. Our study suggests a negative correlation of *Allobaculum* activity with DIO, but not when considering the data obtained by whole community analysis. The *Clostridia* class comprising *Ruminococcus* and *Oscillospira* is closely associated with host energy efficiency and digestibility of carbohydrates (Tims et al., 2013; Salonen et al., 2014). In our study, RNA analysis showed a reduction of *Oscillospira* to be correlated with the lower weight of the HFD+LGG group. It is therefore hypothesized that an indirect reduction of host energy efficiency by LGG may result in a reduced metabolic activity of *Oscillospira* in the gut. Thus, the impact of LGG on calorie consumption apparently affects weight and gut microbiota. LGG is one of the most widely studied probiotics for its functionality, especially with regard to metabolic syndrome (Vajro et al., 2011; Ji et al., 2012; Bull-Otterson et al., 2013; Kim et al., 2013; Lahti et al., 2013; Canani et al., 2016). Kim et al. (2013), for example, reported that LGG improved insulin sensitivity and reduced adiposity in a DIO mice model, together with a significant reduction of SREBP1c in mesenteric adipose tissue as a key biomarker of anti-obesity effects. However, microbiome modulatory effects of LGG have only been rarely reported, and, as far as we know, no study has defined modulation of the active microbiota after LGG treatment.

Administration of probiotics is considered as a promising approach to modulate host microbiota in a beneficial way (Steer et al., 2000; Jia et al., 2008). Anti-obesity effects of probiotics have been reported in animal models (Ji et al., 2012; Park et al., 2013; Wang et al., 2015; Alard et al., 2016) and in clinical trials (Woodard et al., 2009; Kadooka et al., 2010; Kondo et al., 2010). Kadooka et al. (2010) investigated the anti-obesity effect of the probiotic strain *L. gasseri* SBT2055 by conducting a double-blind, randomized, placebo-controlled intervention trial with 87 overweight and obese subjects for 12 weeks. The data confirmed that abdominal visceral and subcutaneous fat, weight, BMI, waist and hip measurements were significantly reduced in the group consuming the probiotic. Larsen et al. (2013) reported fecal microbiota not to be significantly altered in obese adolescent groups after administration of the anti-inflammatory strain *L. salivarius* Ls-33, an observation confirmed in an HFD mouse model (Alard et al., 2016).

We compared metagenome results, respectively, obtained with gDNA and rRNA based analysis. Some of the major groups such as *Bacteroides* spp., *Oscillospira* spp., and *Ruminococcus* spp. were identified as metabolically active microorganisms and are probably responsible for the DIO status of the murine model. Hiergeist et al. (2015) have underlined the key role of the microbiome composition in developmental processes, in host metabolism and physiology, and in different diseases. They have emphasized the importance of experimental protocols and bioinformatics analysis that may have a major impact on the outcome and final interpretation of results. Our data suggest metabolically active Bacteroides to be positively correlated with weight gain.

Characterisation of microbial populations by rRNA data may provide deeper insight in complex ecosystem-related microbial interactions. Yet, extensive experimental data are required to correlate rRNA data both to growth related and non-growth metabolic activities (Blazewicz et al., 2013). This may particularly be applicable to the gut microbial ecosystem and its diverse compartments.

## Author Contributions

YJ, SP, and WH designed the study, and, together with HP and EH, supported the animal trial work and SCFA analysis. YJ and SP prepared the microbiome DNA and RNA and generated 16S amplicons for sequencing and also conducted the bioinformatic analysis of the data. YJ and WH drafted and prepared the manuscript while BP and HS were involved in the editing of the manuscript. All authors approved the final version.

## Conflict of Interest Statement

The authors declare that the research was conducted in the absence of any commercial or financial relationships that could be construed as a potential conflict of interest.
