# Supplementary material for: Modulation of Active Gut Microbiota by Lactobacillus rhamnosus GG in a Diet Induced Obesity Murine Model
Source: Front Microbiol. 2018 Apr 10;9:710. doi: 10.3389/fmicb.2018.00710 (PMC5902571; doi:10.3389/fmicb.2018.00710)
Supplement: Supplementary file 1 [file Data_Sheet_1.PDF]

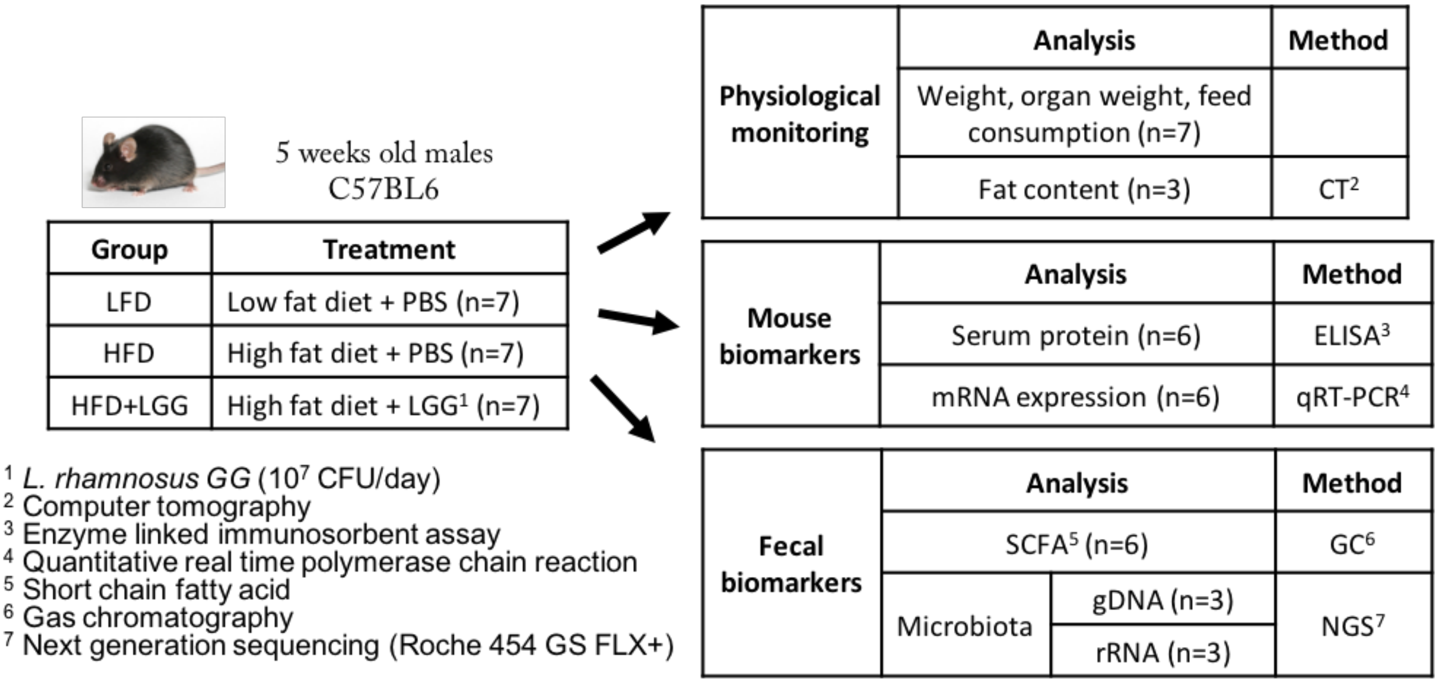

Supplementary Figure S1 Schematic illustration of study design.

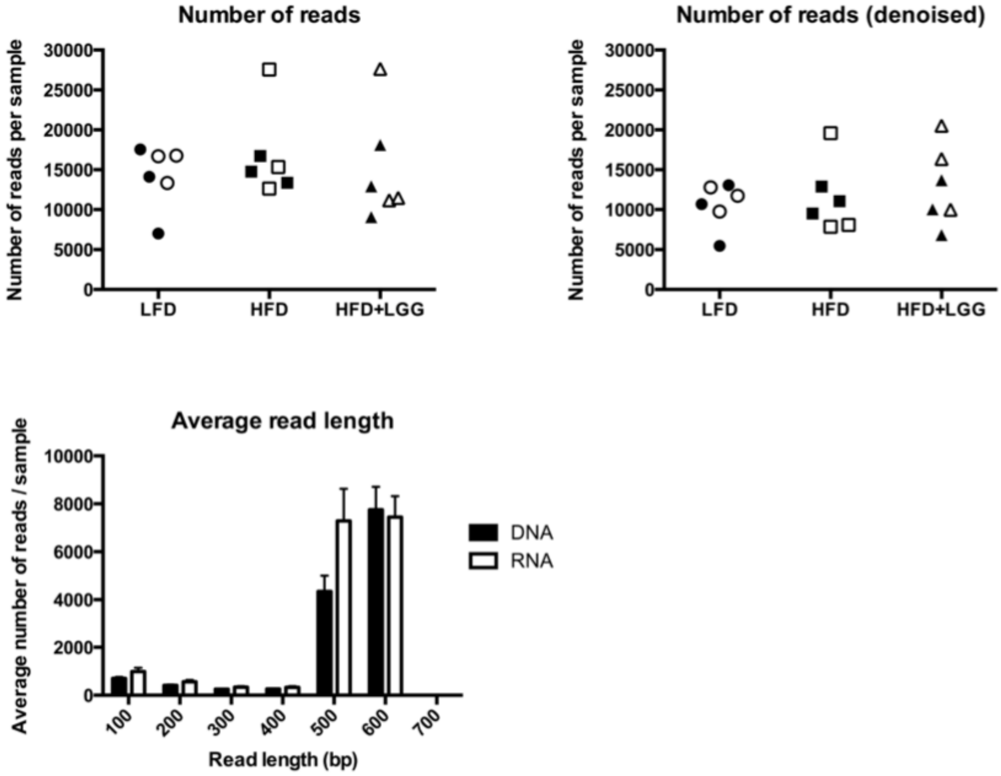

Supplementary Figure S2 Roche 454 GS FLX PLUS was used for metagenomic analysis. (A) total number of reads before and after denoising and chimera removing by FlowClus and Usearch 6.1; (B) Average read length and number of reads per sample depending on DNA and RNA analysis.

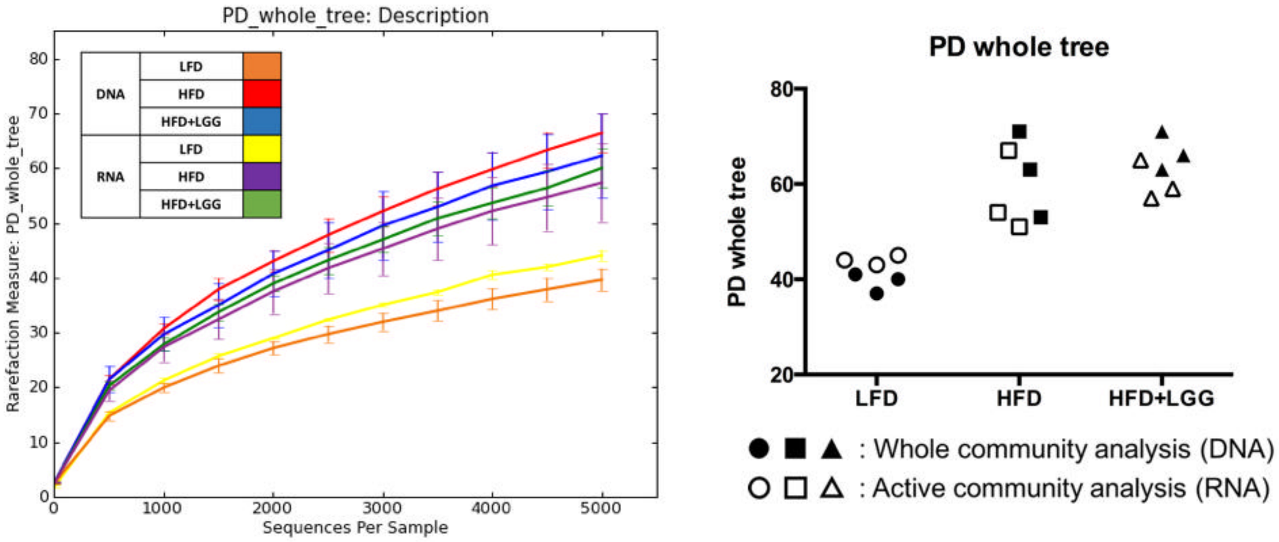

Supplementary Figure S3 PD whole tree rarefaction analysis of different groups

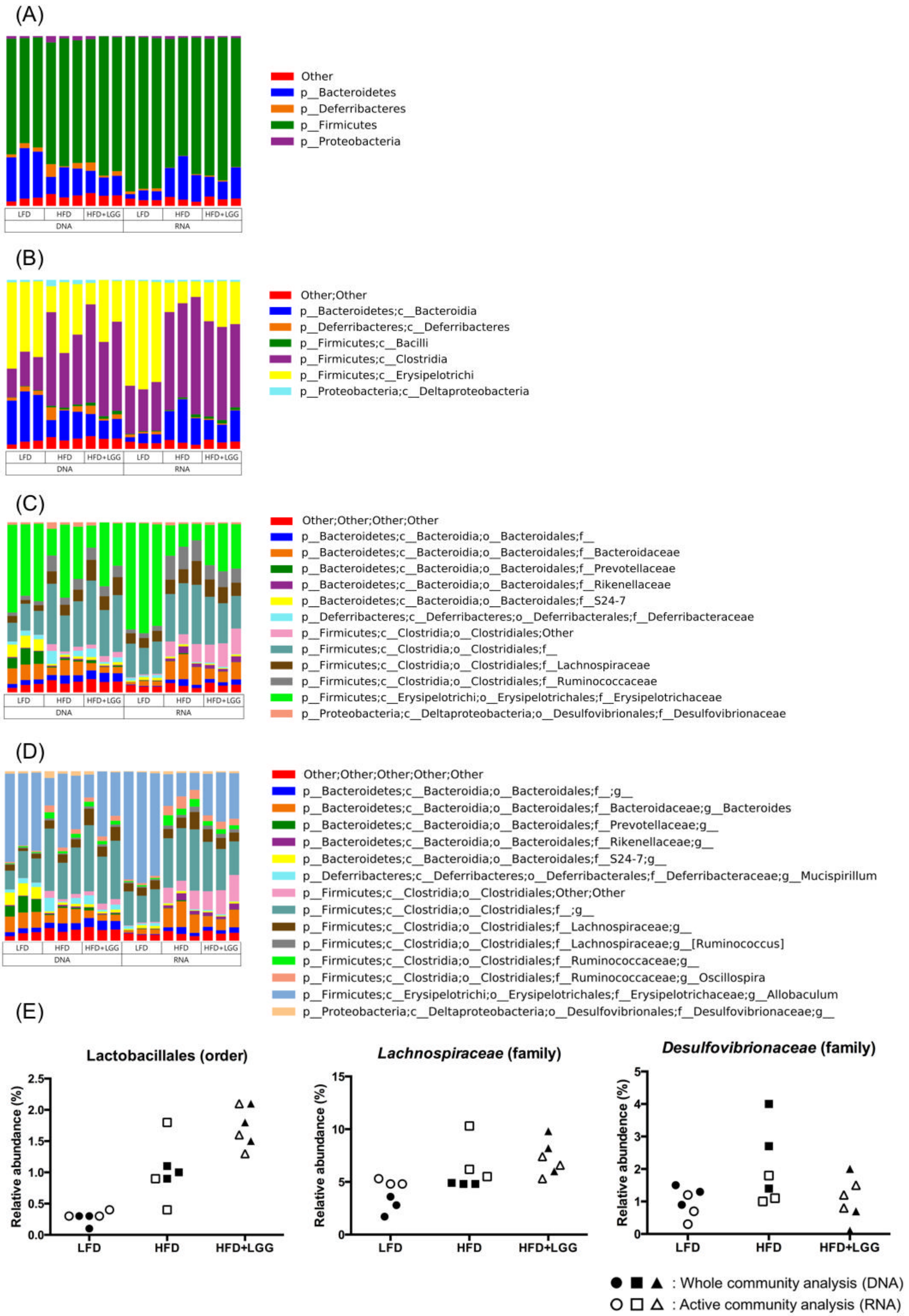

Supplementary Figure S4 QIIME pipeline was used to summarise taxonomic information. (A) Taxonomic summary of phylum level; (B) taxonomic summary of class level; (C) taxonomic summary of family level; (D) taxonomic summary of genus level; (E) Relative abundance of *Lactobacillales*, *Lachnospiraceae* and *Desulfovibrionaceae*.

Supplementary Table 1 List of primers

Target	Sequences	References
FAS	F 5'-CTGGACTCGCTCATGGGTG-3'<br>R 5'-CATTTCCTGAAGTTTCCGCAG-3'	Rascle et al., 2003
SREBP-1c	F 5'-AGCAGCCCCTAGAACAAACAC-3'<br>R 5'-CAGCAGTGAGTCTGCCTTGAT-3'	Xu et al., 2010
CPT1a	F 5'-AAGCCTTTGGGTGGATATGTGA-3'<br>R 5'-ATGGAACTGGTGGCCAATGA-3'	Lin et al., 2011
β-actin	F 5'-CCATCCTGCGTCTGGACTTG-3'<br>R 5'-TTCCCTCTCAGCTGTGGTGG-3'	Lan et al., 2003

Supplementary Table 2 List of barcode sequences

#	Barcode	#	Barcode	#	Barcode
1	ACGAGTGCGT	7	CGTGTCTCTA	13	ATACGACGTA
2	ACGCTCGACA	8	CTCGCGTGTC	14	TCACGTACTA
3	AGACGCACTC	9	TCTCTATGCG	15	CGTCTAGTAC
4	AGCACTGTAG	10	TGATACGTCT	16	TCTACGTAGC
5	ATCAGACACA	11	CATAGTAGTG	17	TGTACTACTC
6	ATATCGCGAG	12	CGAGAGATAC	18	ACGACTACAG

References

Lan, H., Stoehr, J.P., Nadler, S.T., *et al.* (2003). Dimension reduction for mapping mRNA abundance as Quantitative traits. *Genetics* 164, 1607-1614.

Lin, R., Lu, G., Wang, J., Zhang, C., Xie, W., Lu, X., *et al.* (2011). Time course of gene expression profiling in the liver of experimental mice infected with *Echinococcus multilocularis*. *PLoS One* 6, e14557.

Rascle, A., Johnston, J.A., Amati, B. (2003). Deacetylase activity is required for recruitment of the basal transcription machinery and transactivation by STAT5. *Mol Cell Biol* 23, 4162-4173.

Xu, Z., Ouyang, L., Castillo-Olivares, A., *et al.* (2010). α_1-Fetoprotein transcription factor (FTF)/liver receptor homolog-1 (LRH-1) is an essential lipogenic regulator. *Biochim Biophys Acta* 1801, 473-479.
